# MANF clears mutant uromodulin in human kidney organoids of autosomal dominant tubulointerstitial kidney disease

**DOI:** 10.1172/JCI205631

**Published:** 2026-08-25

**Authors:** Chenjian Gu, Yili Fang, Yixuan Wang, Eric Tycksen, Gayathri Kondepati, Chuang Li, Kendrah O. Kidd, Jun Liu, Fumihiko Urano, Maria Lindahl, Anthony J. Bleyer, Srikanth Singamaneni, Zhao Sun, Ying Maggie Chen

**Affiliations:** 1 Department of Medicine,; 2 Institute of Materials Science and Engineering, and; 3 Genome Technology Access Center, Washington University School of Medicine, St. Louis, Missouri, USA.; 4 Department of Medicine, Wake Forest University, Winston-Salem, North Carolina, USA.; 5 Shanghai General Hospital, Shanghai, China.; 6 University of Helsinki, Helsinki, Finland.; 7 Department of Biological Sciences, The University of Texas at Dallas, Richardson, Texas, USA.; 8 St. Louis Veteran Affairs Medical Center, St. Louis, Missouri, USA.

**Keywords:** Genetics, Nephrology, Autophagy, Calcium signaling, Molecular genetics

## Abstract

In human kidney organoids, MANF treats genetic kidney disease ADTKD-*UMOD* by blocking ER calcium leakage and restoring autophagy to clear the toxic mutant protein.

**To the Editor:** Autosomal dominant tubulointerstitial kidney disease due to *UMOD* mutations (ADTKD-*UMOD*) is a leading monogenic cause of chronic kidney disease (CKD), and *UMOD* p.H177-R185del is the most prevalent pathogenic variant in the US, accounting for 21% of cases. Currently, therapy is unavailable. UMOD is synthesized and secreted by the thick ascending limb (TAL) of Henle’s loop. We generated an ADTKD mouse model carrying the human H177-R185del-equivalent deletion mutation and demonstrated that mutant UMOD-induced ER stress suppresses p-AMPK-FOXO3-mediated autophagy in TALs. Moreover, overexpression of mesencephalic astrocyte-derived neurotrophic factor (MANF), an ER chaperone, ameliorates CKD by enhancing p-AMPK in mice (1).

To bolster translational relevance and delineate how MANF activates p-AMPK, induced pluripotent stem cells (iPSCs) from a heterozygous H177-R185del (*UMOD* ^DEL/+^) patient and an unaffected family member (Supplemental Figure 1A; supplemental material available online with this article; https://doi.org/10.1172/JCI205631DS1) were differentiated into kidney organoids containing TALs (Figure 1, A and B), proximal tubules, and collecting ducts (Supplemental Figure 1, B and C). The under-glycosylated mutant UMOD localized to the ER with diffuse intratubular expression, whereas WT UMOD displayed apical TAL enrichment (Figure 1B). Furthermore, *UMOD* ^DEL/+^ TALs isolated from organoids recapitulated hallmarks of our ADTKD mice, including ER stress activation (BiP and p50ATF6 induction) and impaired autophagy (p-AMPK/FOXO3 inhibition and p62/LC3B-II accumulation) (Figure 1C). In the mutant TALs, RNA-seq showed upregulation of TAL injury markers, as identified in the Human Kidney Atlas (HKAv2) (2), and inflammation as the most upregulated pathway (Supplemental Figure 1, D and E).

Given the interplay between ER stress and calcium dynamics, secreted ER-calcium-monitoring protein–expressing (SERCaMP-expressing) (3) WT or DEL/+ hiPSCs were utilized, and differentiated DEL/+ organoids exhibited marked ER calcium leakage (Figure 1D). Examination of key ER calcium release channels revealed selective increase of inositol 1,4,5-trisphosphate receptor 1 (IP3R1) at both transcriptional and translational levels, but not IP3R2, IP3R3, and ryanodine receptors (RyRs) in the mutant TALs (Figure 1, E–G). We next investigated whether tribbles homolog 3 (TRIB3), an ER stress–inducible pseudokinase, is implicated following ER calcium leak. Dual-immunofluorescence (dual-IF) staining and IB showed that the pronounced induction of TRIB3 in DEL/+ TALs (Figure 1, H and I) was attenuated by the IP3R blocker Xestospongin C (XeC) (Supplemental Figure 2A), indicating TRIB3 as a downstream effector of ER calcium depletion.

Since AMPK links stress responses and autophagy promotion, we further determined whether TRIB3 could modulate AMPK activity. Co-IP revealed that endogenous TRIB3 directly binds to the catalytic subunit AMPKα in the organoid-isolated TALs (Figure 1J). Deletion mapping of FLAG-tagged AMPKα1/α2 demonstrated a direct interaction between TRIB3 and the activation loop (AL) of both AMPKα1/α2 (Supplemental Figure 2, B–D), which may block T172/T183 phosphorylation within the AL. In stable HEK293 cells expressing WT or *UMOD* H177-R185del, TRIB3 was induced and p-AMPKα (T172/T183) was reduced in DEL cells as well (Supplemental Figure 2E). Knockdown and overexpression of TRIB3 further validated that TRIB3 negatively regulated AMPKα phosphorylation (Supplemental Figure 2, E and F). Collectively, these data demonstrate that IP3R1-mediated ER calcium depletion, triggered by misfolded UMOD-induced ER stress, upregulates TRIB3, which can directly bind to and suppress AMPK activation, thereby impairing autophagy.

To determine whether MANF exerts a therapeutic role in ADTKD organoids, tetracycline-inducible MANF-overexpressing (TRE-MANF) iPSCs were generated (Supplemental Figure 3A) and differentiated into kidney organoids. Upon doxycycline induction from days 22–30, MANF overexpression in the DEL/+ organoids, including TALs (Figure 1K and Supplemental Figure 3B) substantially mitigated IP3R1 expression (Figure 1K and Supplemental Figure 3C) through direct interaction with IP3R1 (Figure 1L), thus attenuating ER calcium depletion (Figure 1M). The subsequent TRIB3 repression (Figure 1N and Supplemental Figure 3D) and restoration of p-AMPK/FOXO3 expression by MANF upregulation (Figure 1N) promoted autophagic flux (Supplemental Figure 3E) and clearance of mutant UMOD (Figure 1O), thereby ameliorating ER stress, tubular injury, and apoptosis, as well as enhancing WT UMOD secretion (Figure 1P and Supplemental Figure 3F). Additionally, ultrasensitive plasmon-enhanced fluorescence-linked immunosorbent multiplex assay (p-FLISA) developed in house (4) (Supplemental Figure 3G) showed that MANF overexpression inhibited proinflammatory cytokine secretion (Figure 1Q) and their concordant synthesis (Supplemental Figure 3H) in the mutant TALs. Consequently, enhanced MANF expression led to mutant TAL recovery, as demonstrated by increased expression of healthy TAL genes (HKAv2) (Figure 1R).

Importantly, MANF-overexpressing lentivirus treatment of DEL/+ organoids, from days 22–30, blocked IP3R1-mediated ER calcium depletion and subsequently relieved TRIB3-induced p-AMPK suppression and defective autophagic degradation of mutant UMOD (Supplemental Figure 4, A–E). Correspondingly, the lentivirus treatment reproduced the protective effects of lentiviral vector–mediated MANF upregulation in DEL/+ hiPSCs (Supplemental Figure 4, F–H).

In summary, we discovered that MANF restores compromised autophagy by blunting ER-calcium efflux in ADTKD organoids (Supplemental Figure 4I). By targeting IP3R1, MANF may emerge as a potent therapy to correct disrupted proteostasis in ADTKD and other protein misfolding diseases. Additionally, our ADTKD organoids provide a human-relevant platform for high-throughput therapeutic screening, including RNA-based therapies.

## Conflict of interest

SS is a cofounder and shareholder of Brightest Bio. FU receives royalties from Novus and Sana Biotechnology; licensing/consulting fees from Opris and Emerald Biotherapeutics; and holds patent 9,891,231.

## Funding support

This work is the result of NIH funding in part, and is subject to the NIH Public Access Policy. Through acceptance of this federal funding, the NIH has been given the right to make the work publicly available in PubMed Central.

- American Society of Nephrology (ASN) Kidney Cure Ben J. Lipps Research Fellowship Program (to CJG).
- American Heart Association (AHA) Postdoctoral Fellowship (to YLF).
- NIH R01 DK132090 and P30 DK020579 (to FU).
- 17-2013-410 and 2-SRA-2018-496-A-B from Juvenile Diabetes Research Foundation (to ML).
- Grants from the Finnish Diabetes Research Foundation (to ML).
- 117044 and 333974 from Academy of Finland (to ML).
- NIH R21DK106584 (to AJB).
- 75F40124C00106, Subaward Critical Path Institute FDA contract (to AJB).
- CBET-2316285 and CBET-2331330, National Science Foundation (to SS).
- HT94252310996, Congressionally Directed Medical Research Programs (to SS).
- NIH R01 2DK105056-6, R56DK138158A1, R21DK131557A1, R03DK106451 and K08DK089015 (to YMC).
- VA Merit I01BX006401A1 (to YMC).
- The Office of the Assistant Secretary of Defense for Health Affairs through the Peer Reviewed Medical Research Program under Award W81XWH-19-1-0320 (to YMC).
- NIH P30 DK114857 and NIH P30 DK079337, George M. O’Brien Kidney Research Core Centers (to YMC).
- Seed Grant from WashU Center of Regenerative Medicine and Mallinckrodt Challenge Grant (to YMC).
- Investigator Matching Micro Grant from WashU Center for Drug Discovery (to YMC).
- Faculty Scholar Award from the Children’s Discovery Institute of WashU and St. Louis Children’s Hospital (to YMC).
- NCI Cancer Center Support Grant #P30 CA91842 to the Siteman Cancer Center from the National Center for Research Resources (NCRR), a component of the National Institutes of Health (NIH), and NIH Roadmap for Medical Research (The Genome Technology Access Center at the McDonnell Genome Institute).

## Supplementary Material

Supplemental data

Unedited blot and gel images

Supporting data values

## Figures and Tables

**Figure 1:**
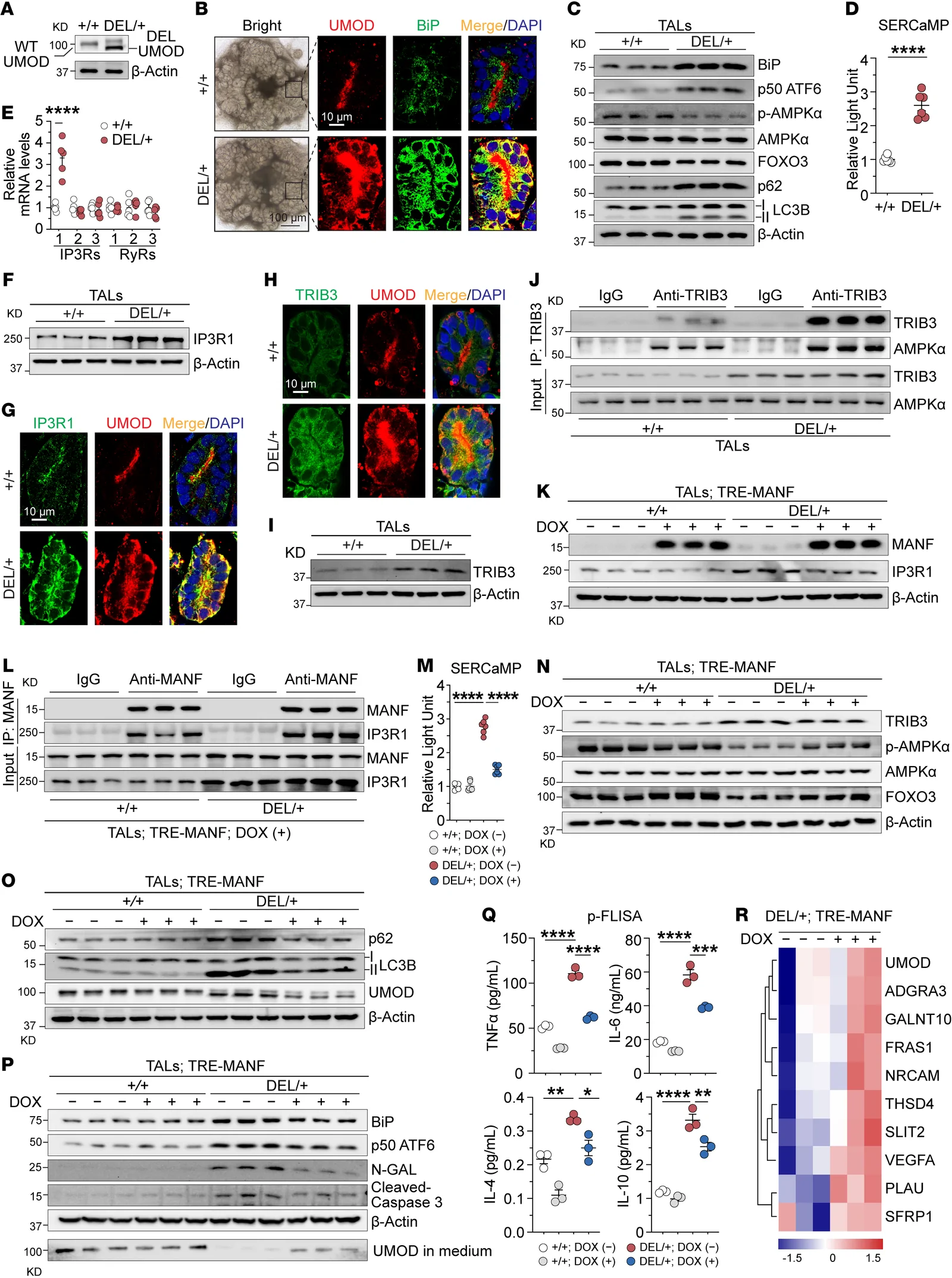
MANF promotes UMOD proteostasis by regulating IP3R1-mediated ER calcium efflux in ADTKD kidney organoids. (**A**) WB of UMOD in organoids. (**B**) Bright-field or double-IF of UMOD and BiP with DAPI in organoids. (**C**, **F**, and **I**) IB of indicated proteins in organoid-isolated TALs. (**D**) Luciferase activity in the organoid medium. (**E**) Transcript expression of ER calcium efflux channels in organoid-derived TALs. (**G**) IF of IP3R1 and UMOD. (**H**) Dual-IF staining of TRIB3 and UMOD. (**J**) Co-IP demonstrating the association of TRIB3 and AMPKα in TALs. (**K**–**R**) Organoids were treated without or with DOX from days 22–30. (**K** and **N**–**P**) IB of the indicated proteins in TALs. (**L**) Co-IP showing the interaction between MANF and IP3R1 in TALs with DOX. (**M**) Luciferase activity in the organoid medium. (**Q**) p-FLISA of cytokines in the organoid medium. (**R**) Heatmap of TAL healthy state markers. Benjamini-Hochberg adjusted *P* < 0.05. *P < 0.05; **P < 0.01; ***P < 0.001; ****P < 0.0001.

## Version 1 08/25/2026

In-Press Preview

## Version 2 10/15/2026

Electronic publication
