# Supplementary material for: MANF clears mutant uromodulin in human kidney organoids of autosomal dominant tubulointerstitial kidney disease
Source: J Clin Invest. 2026 Aug 25;136(20):e205631. doi: 10.1172/JCI205631 (PMC13668634; doi:10.1172/JCI205631)

Figure 1A

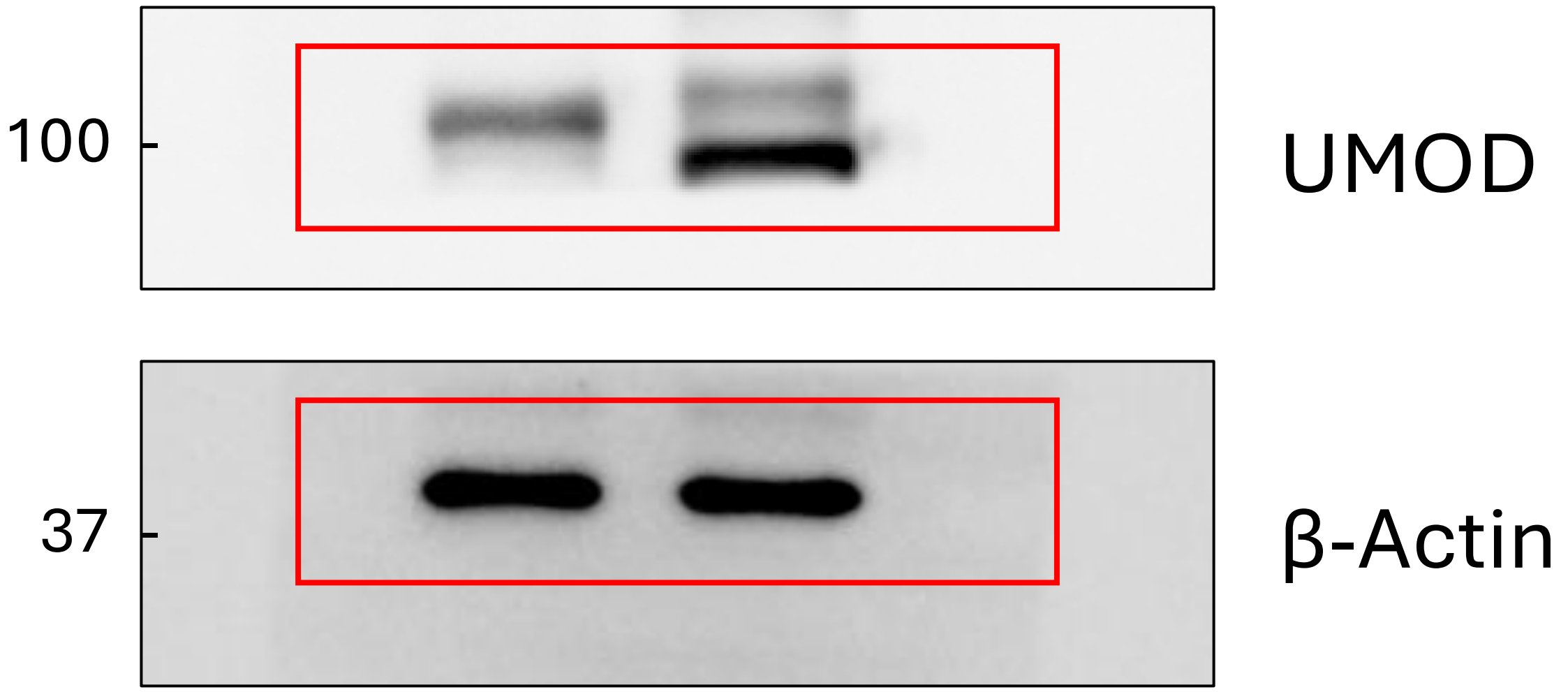

Figure 1C

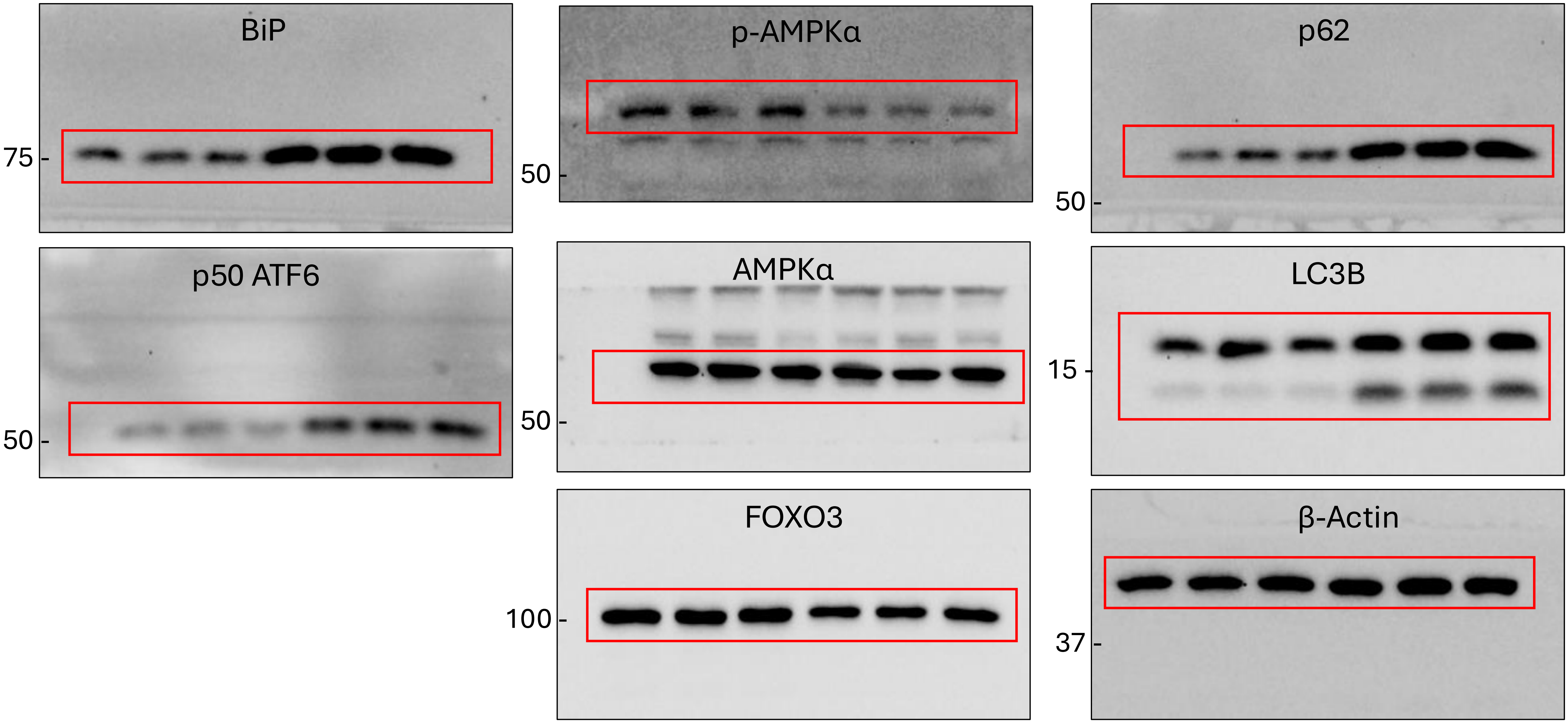
BiP
75 -
p50 ATF6
50 -
p-AMPKα
50 -
AMPKα
50 -
FOXO3
100 -
p62
50 -
LC3B
15 -
β-Actin
37 -

Figure 1F

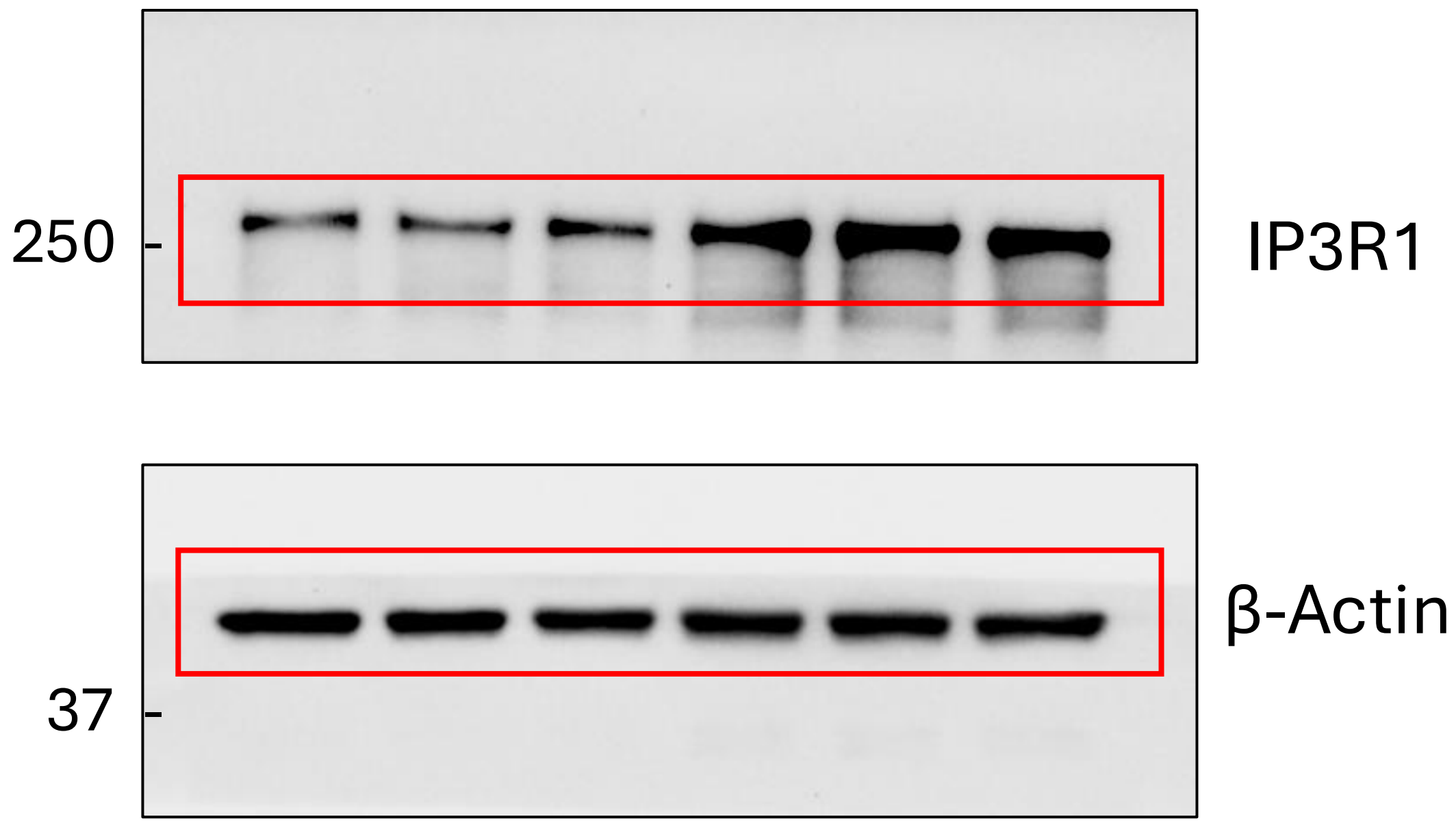

Figure 1I

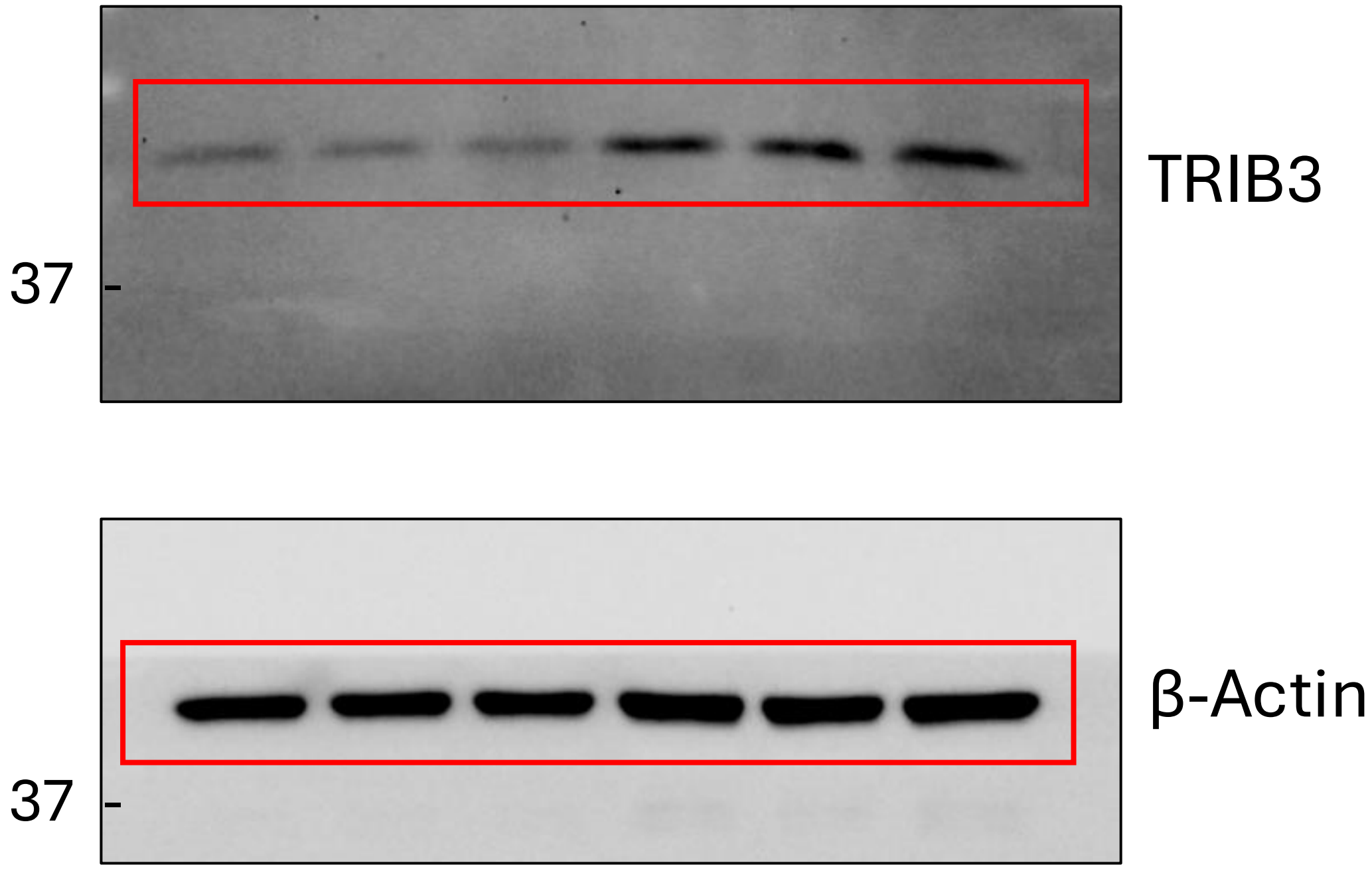

Figure 1J

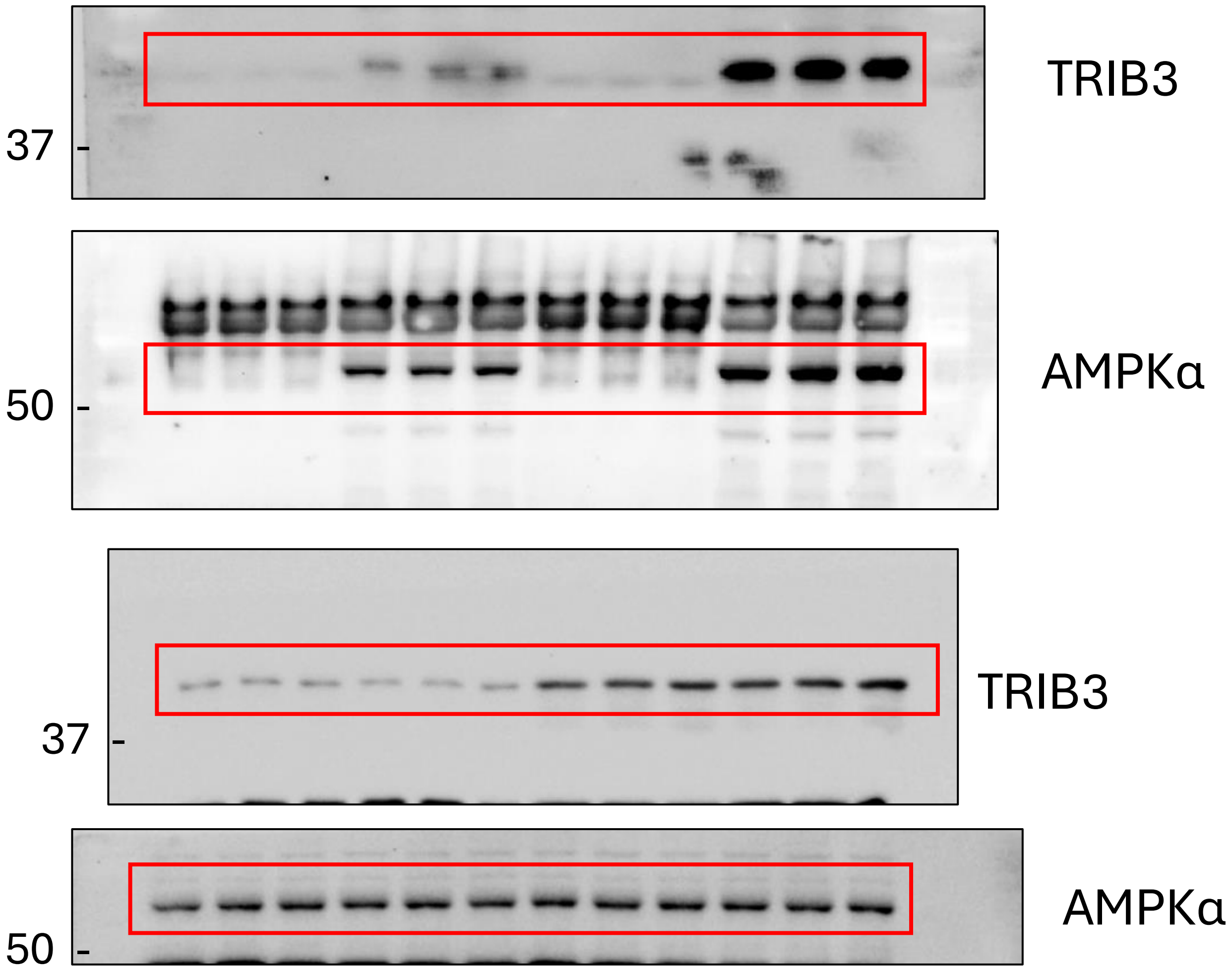

Figure 1K

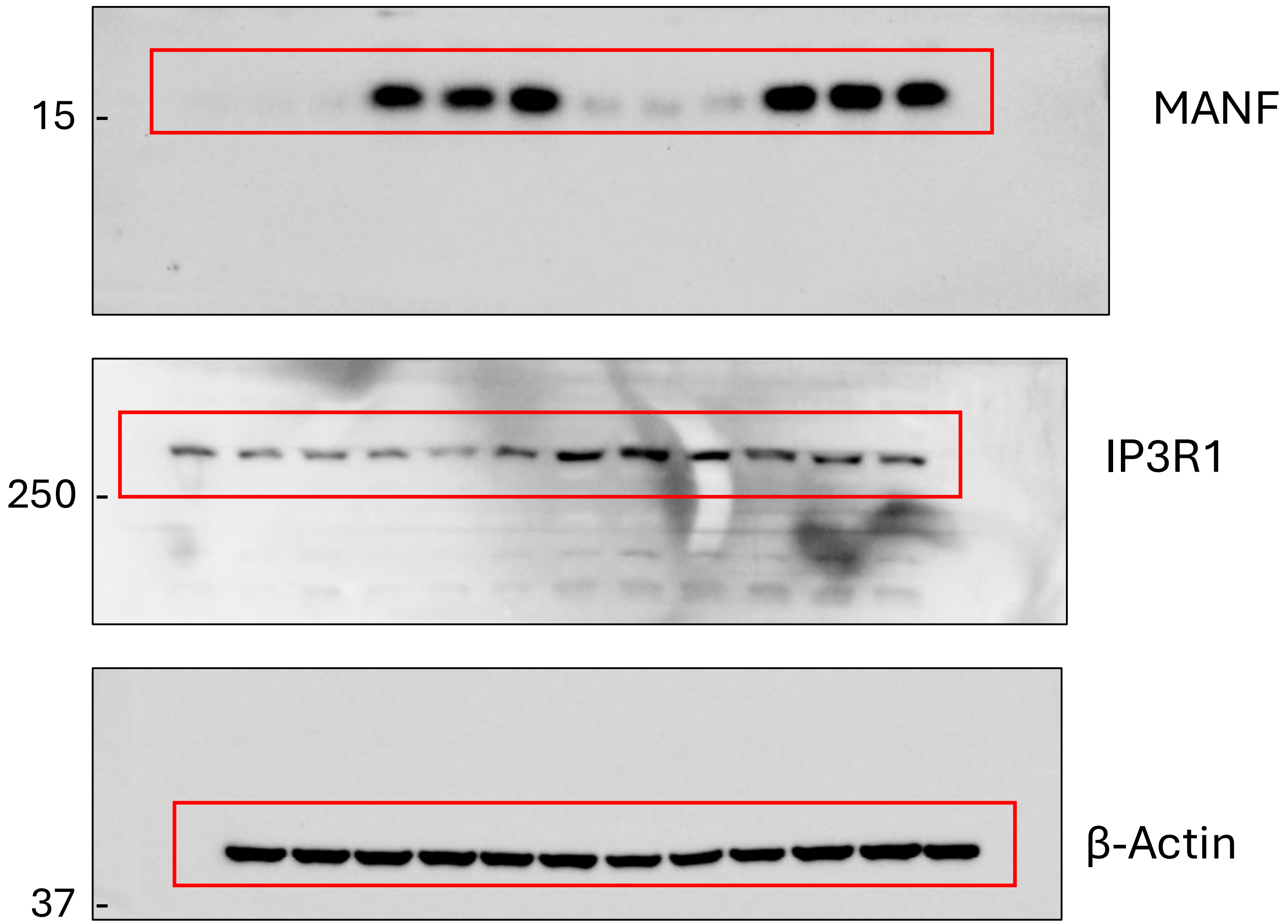

Figure 1L

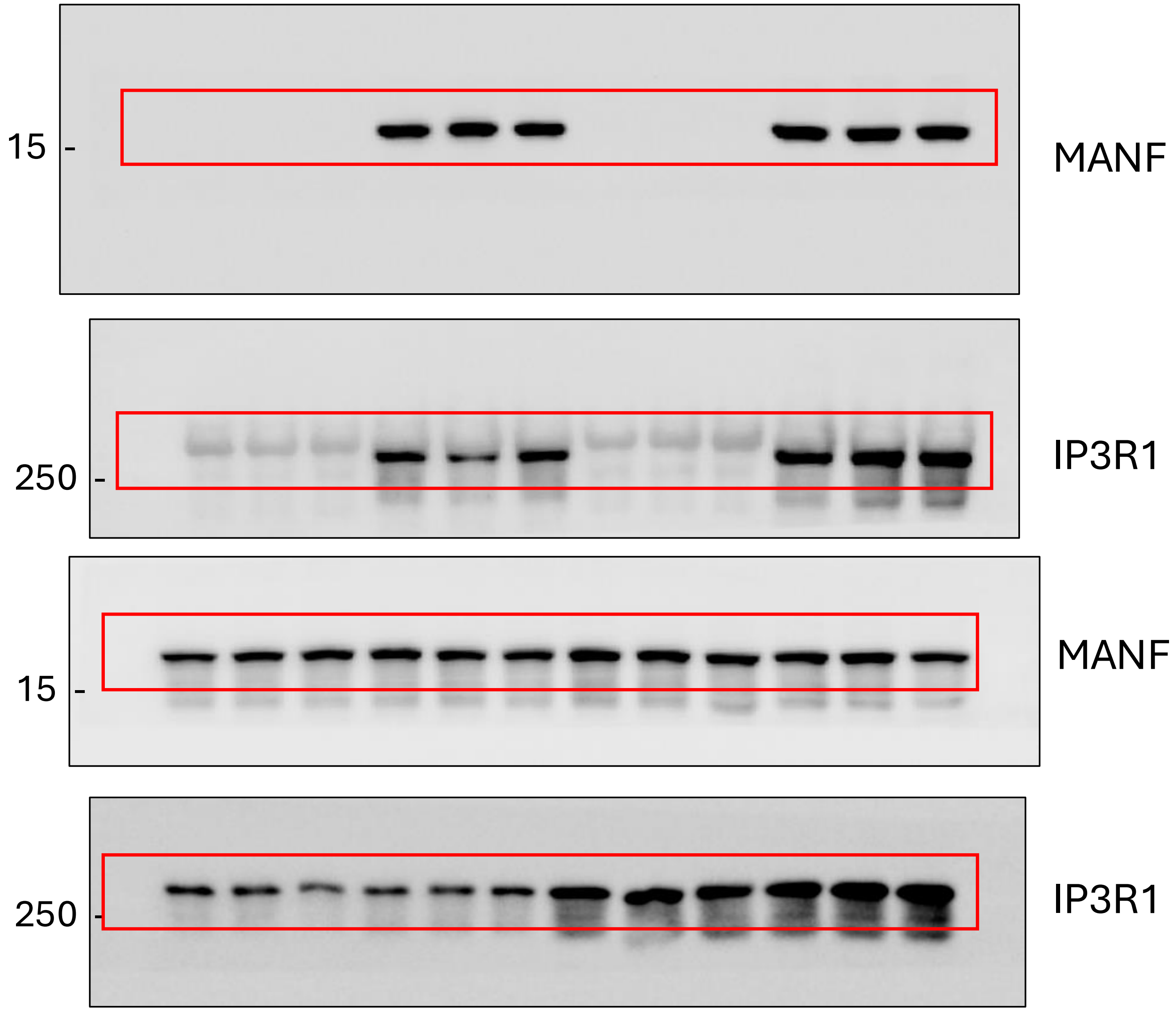

Figure 1N

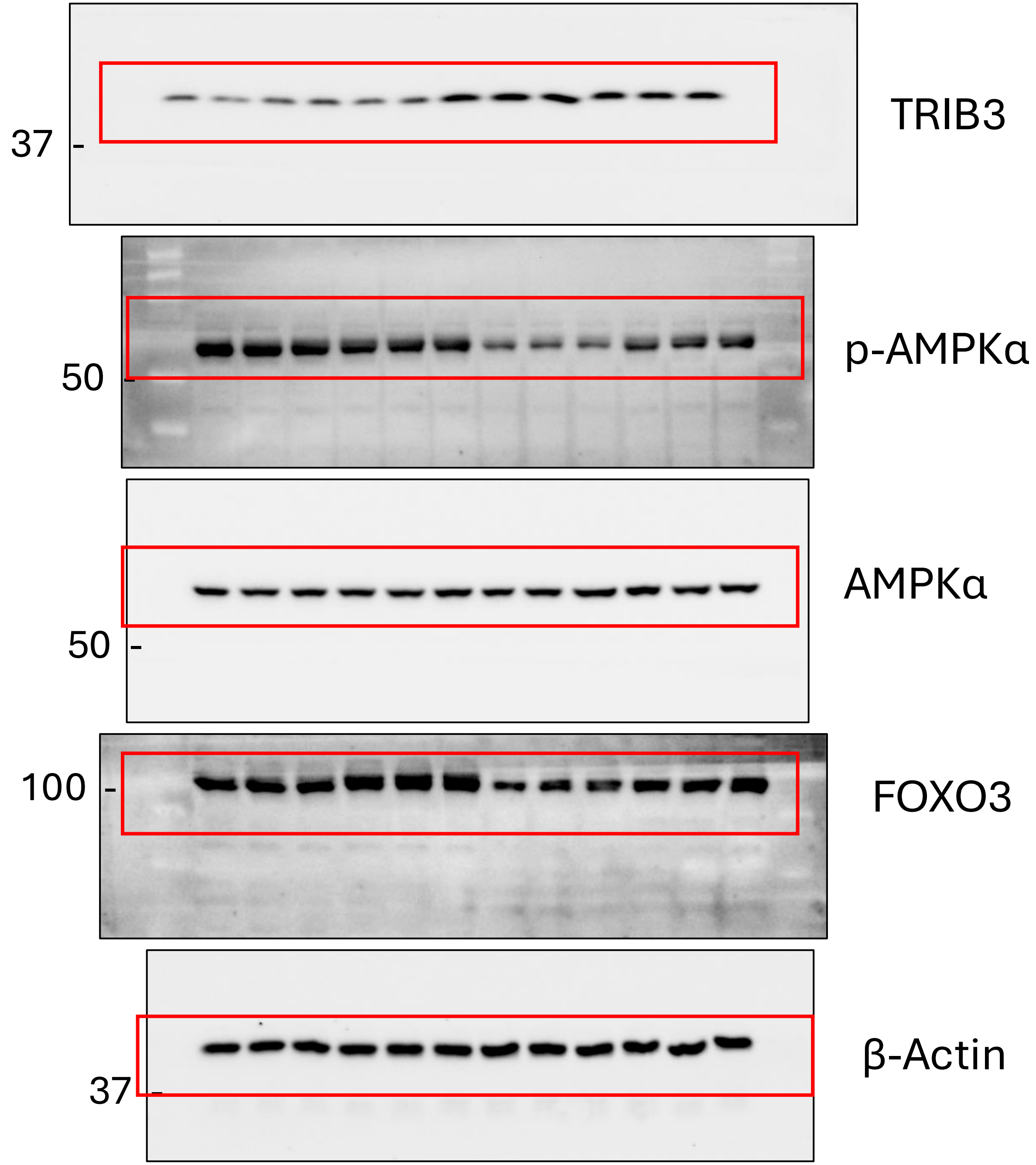

Figure 10

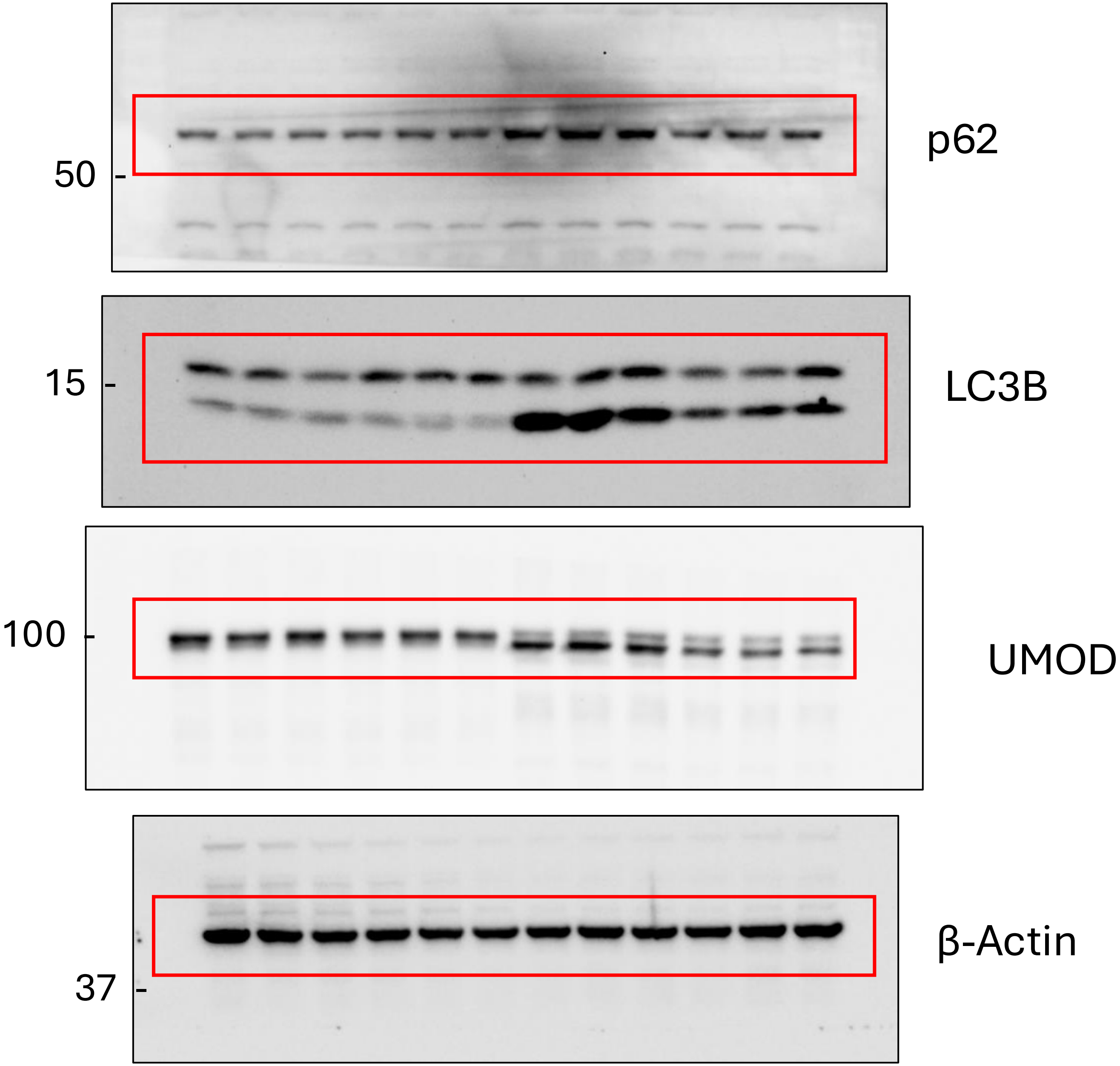

Figure 1P

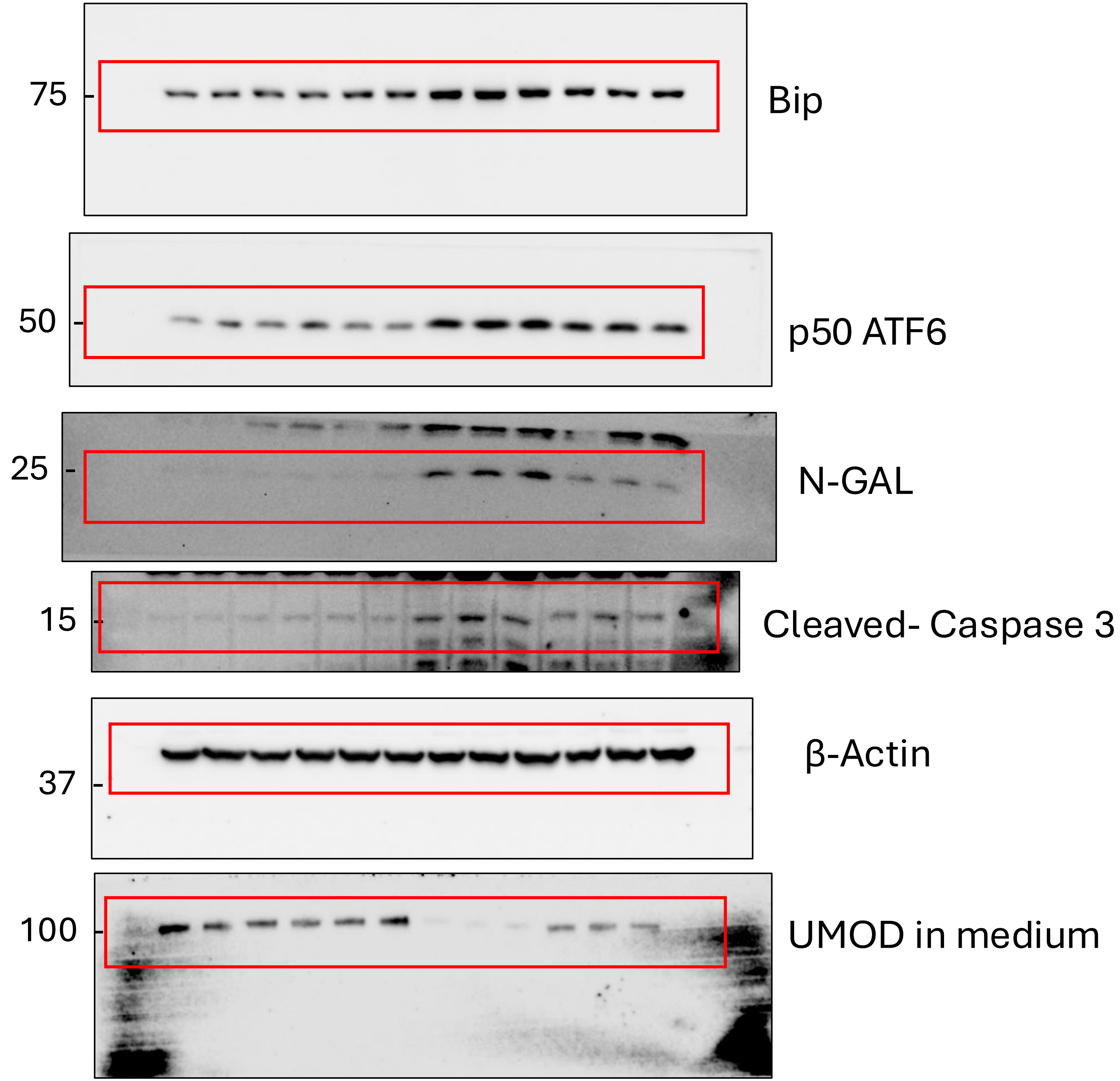

75
Bip
50
p50 ATF6
25
N-GAL
15
Cleaved- Caspase 3
37
β-Actin
100
UMOD in medium

Supplementary Figure 1A

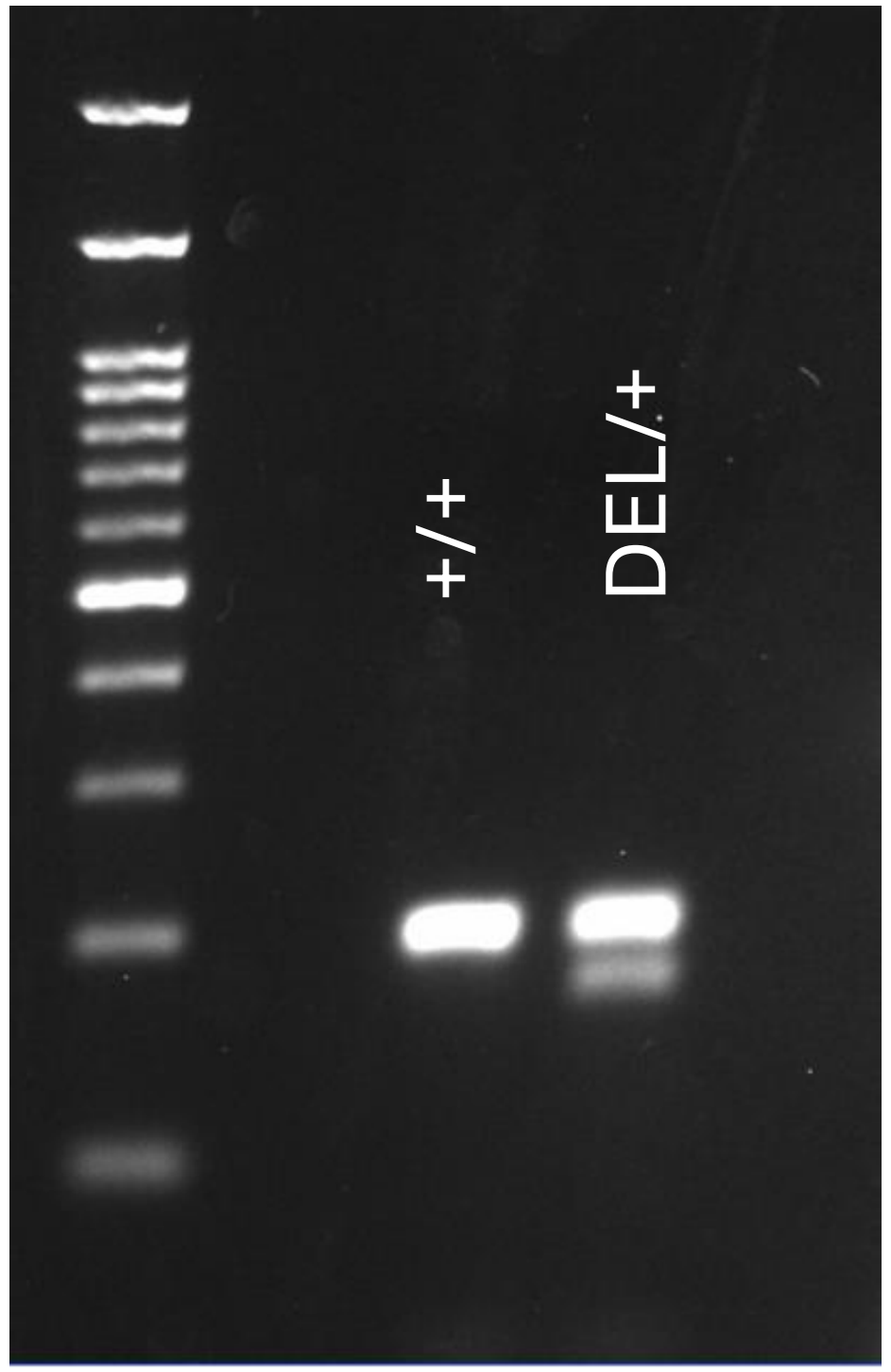

Supplementary Figure 2A

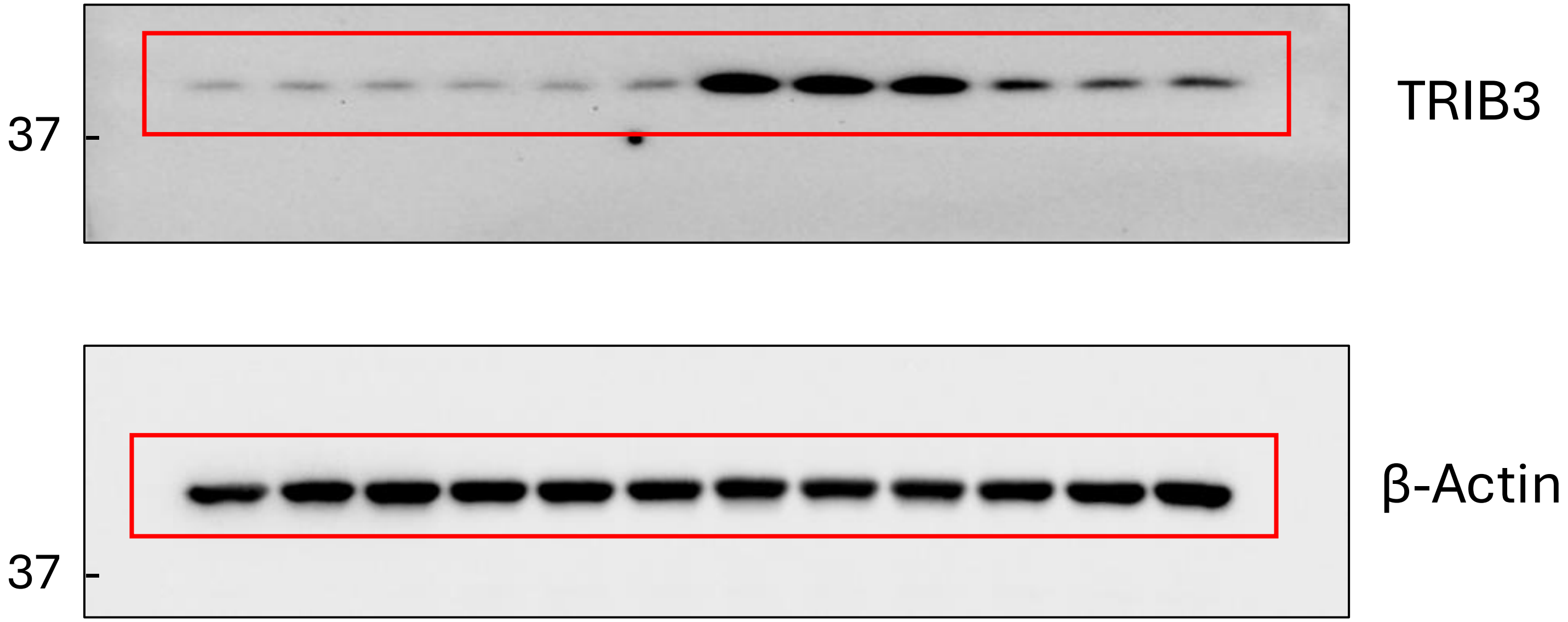

Supplementary Figure 2C

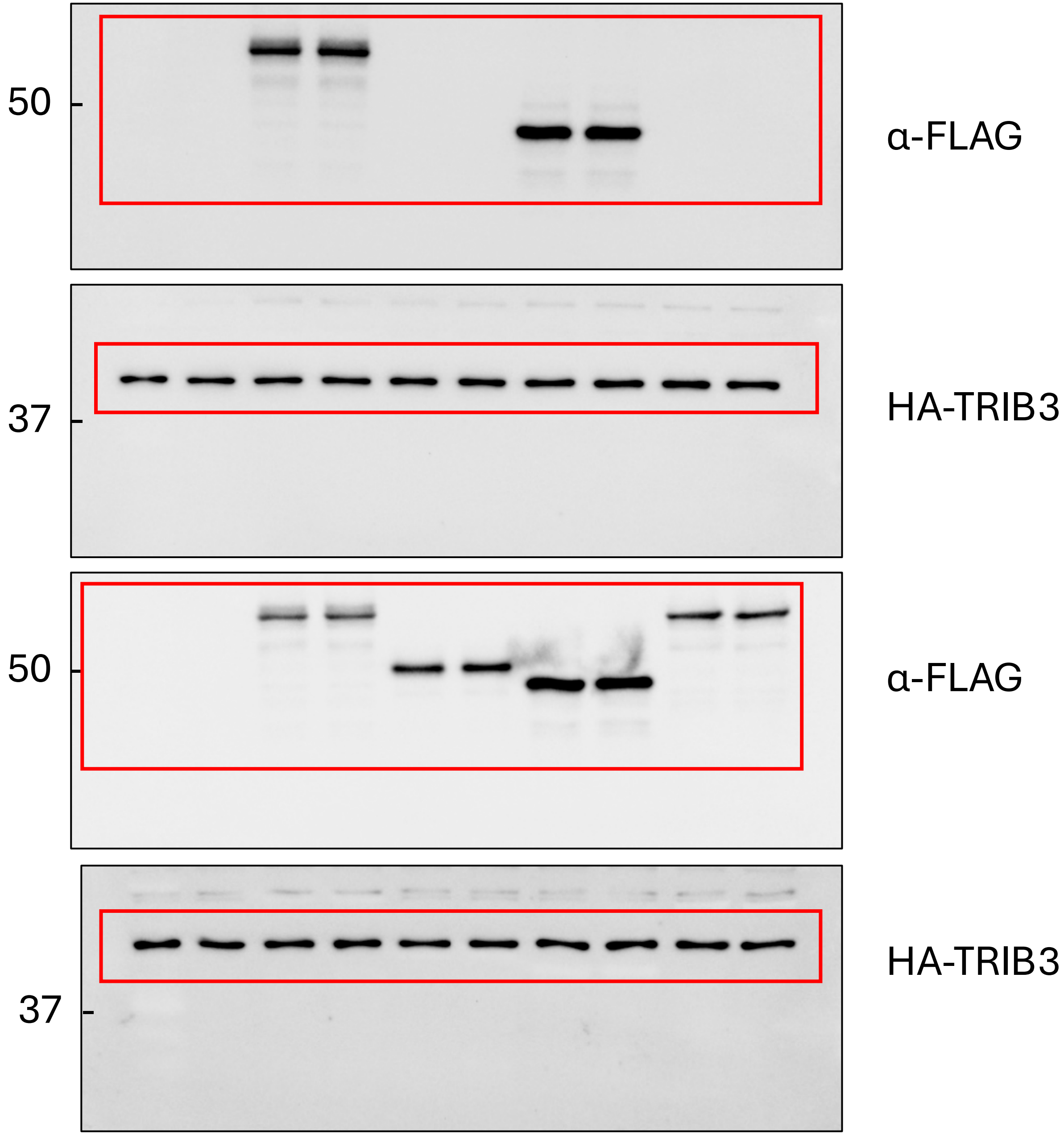

Supplementary Figure 2D

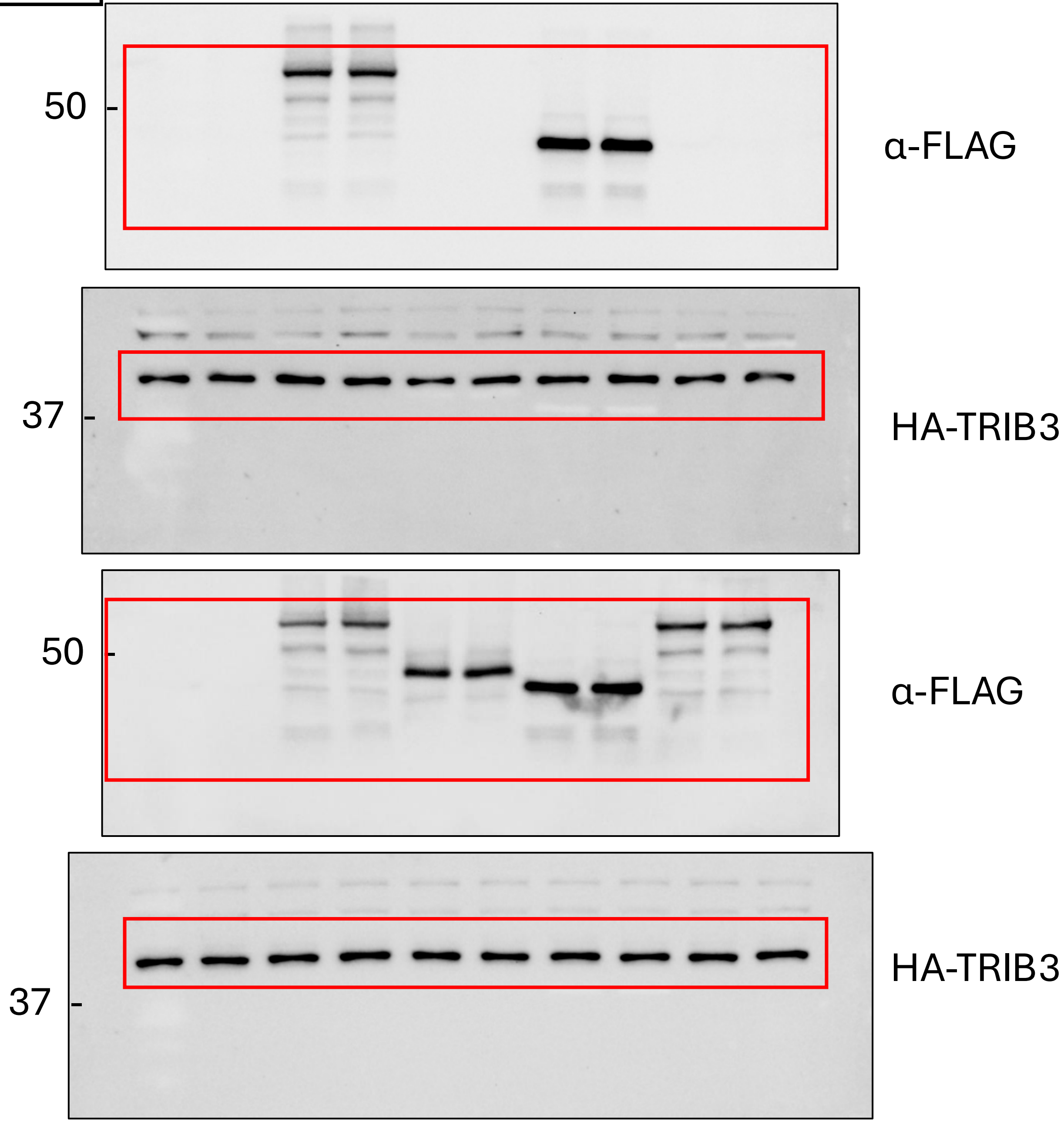

Supplementary Figure 2E

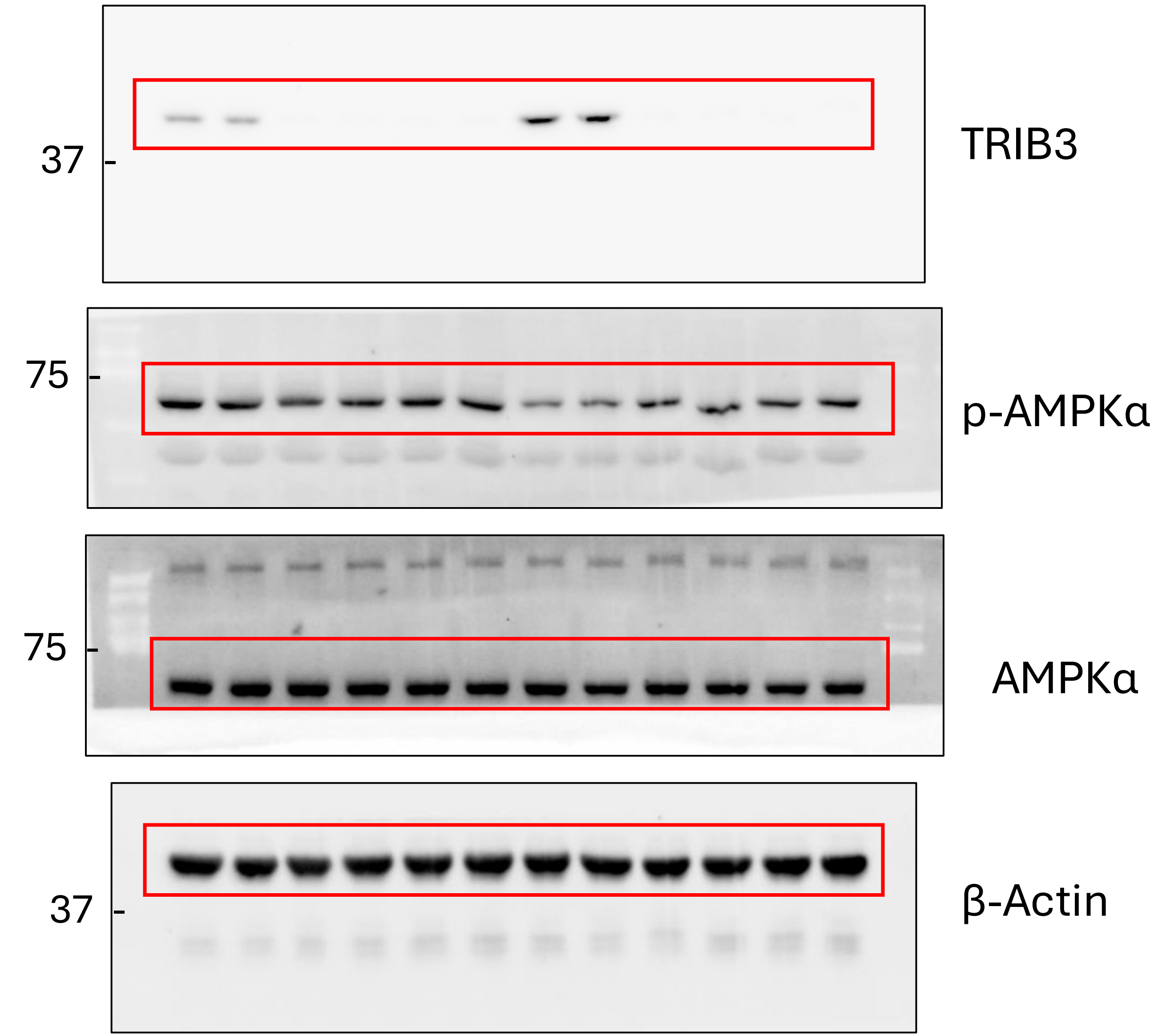

Supplementary Figure 2F

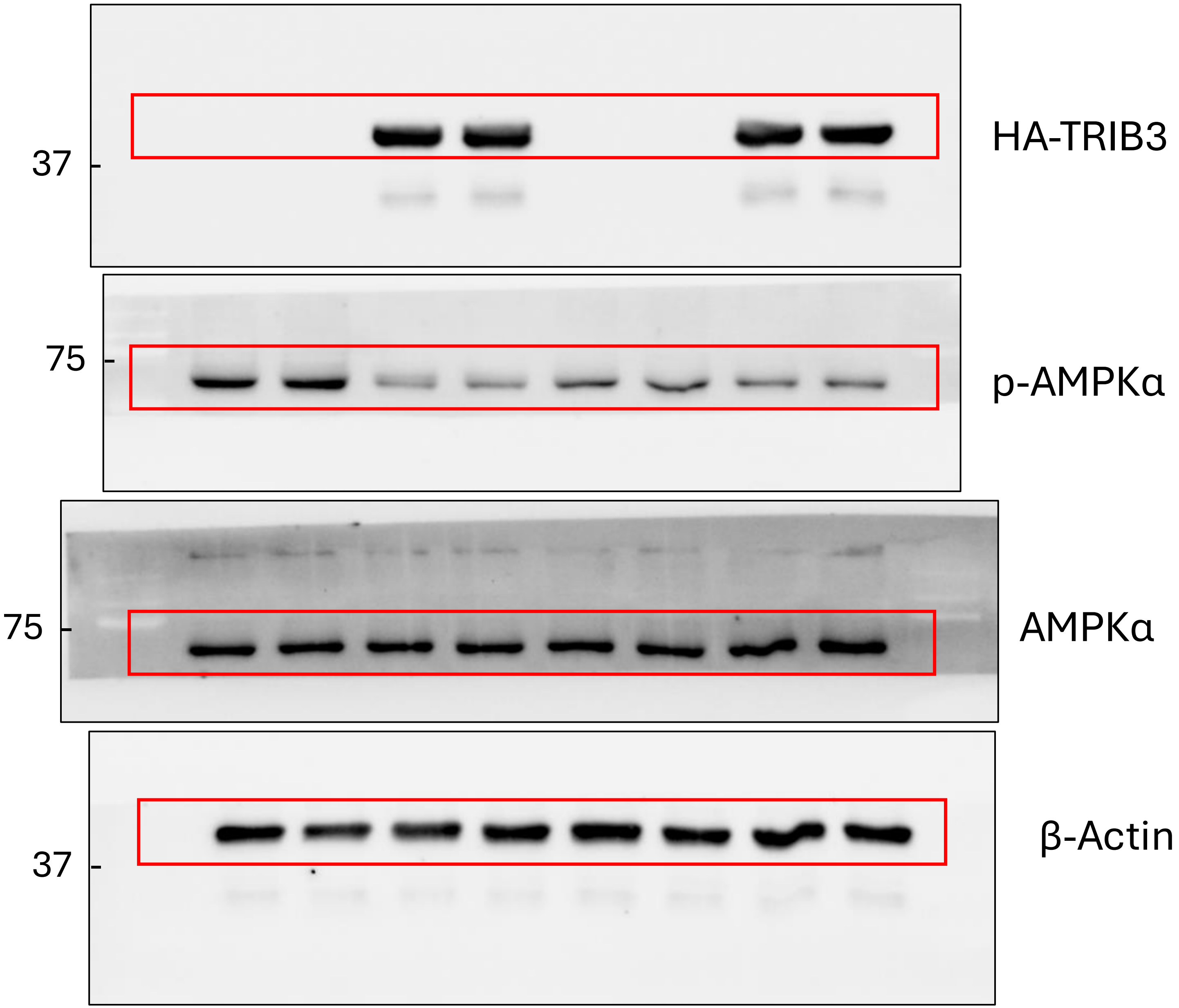

Supplementary Figure 4A

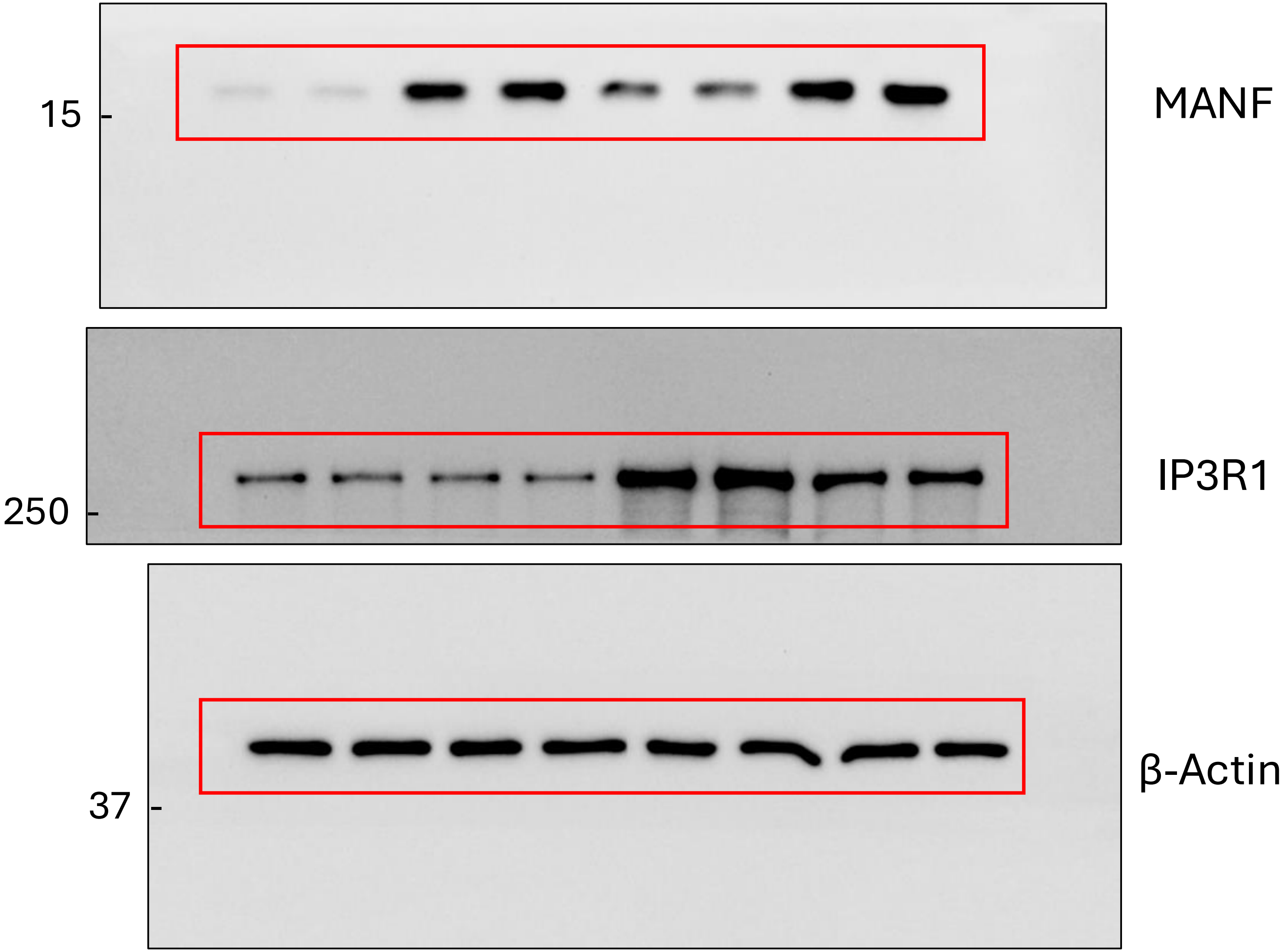

Supplementary Figure 4C

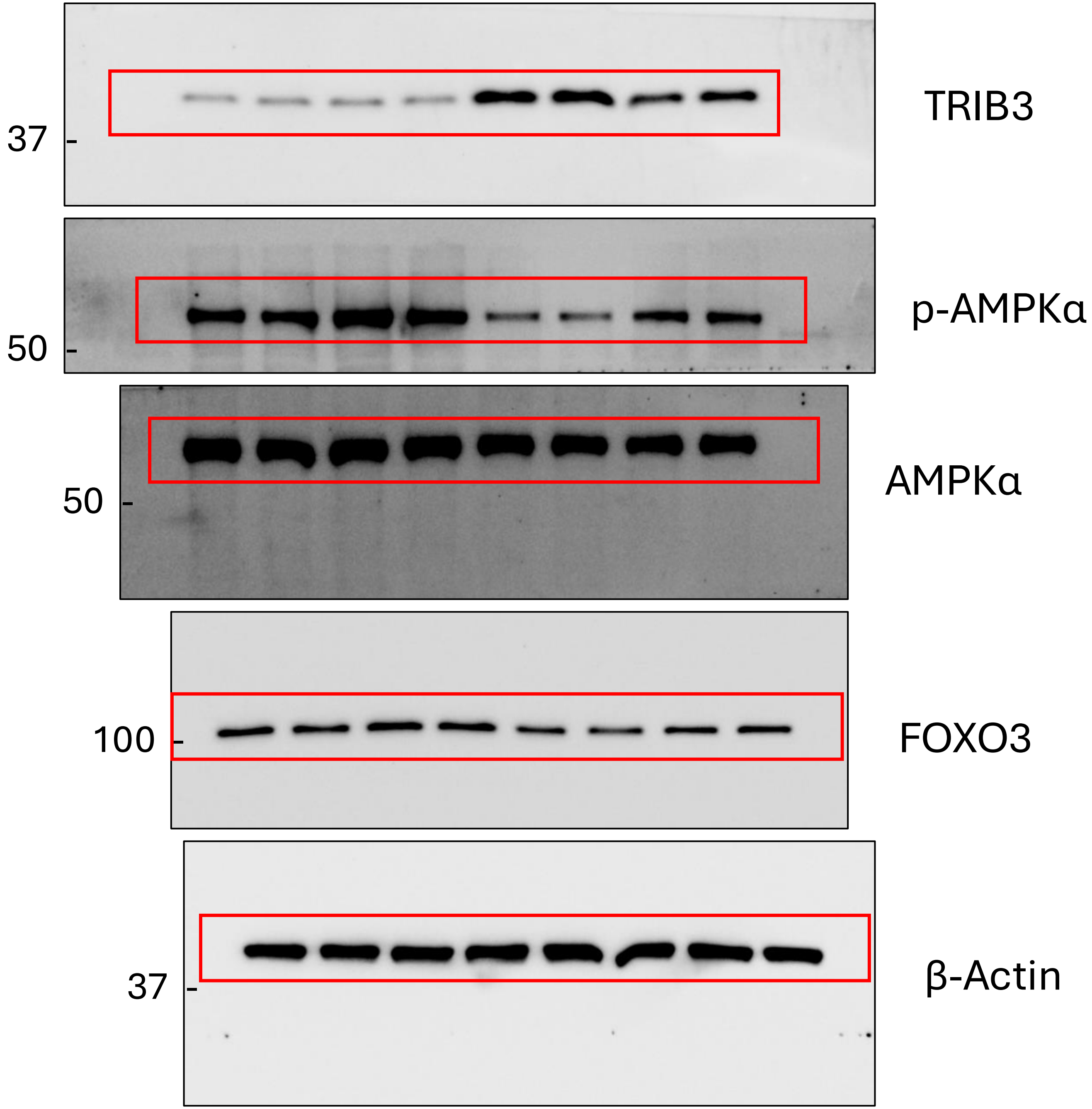

Supplementary Figure 4E

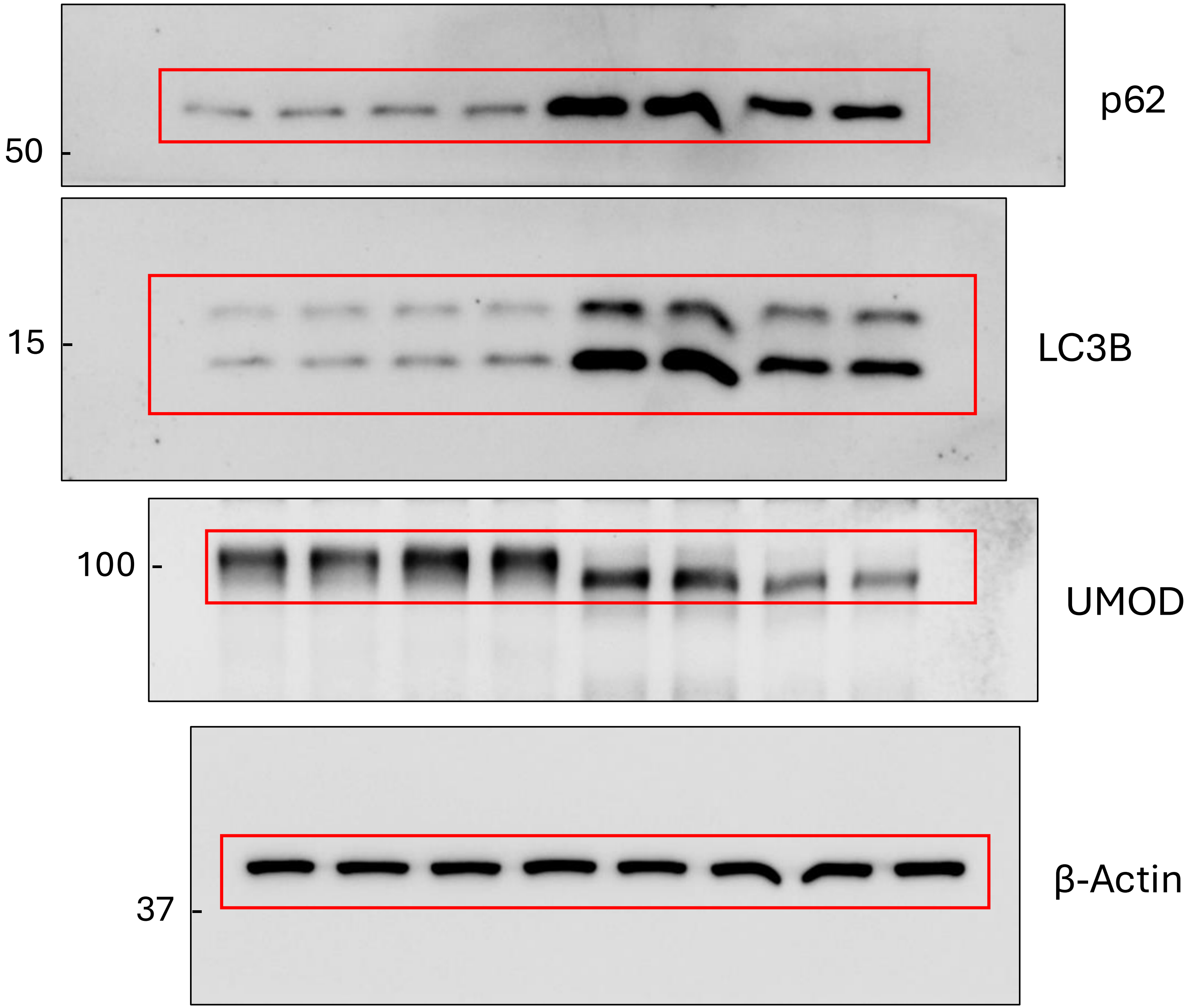

Supplementary Figure 4F

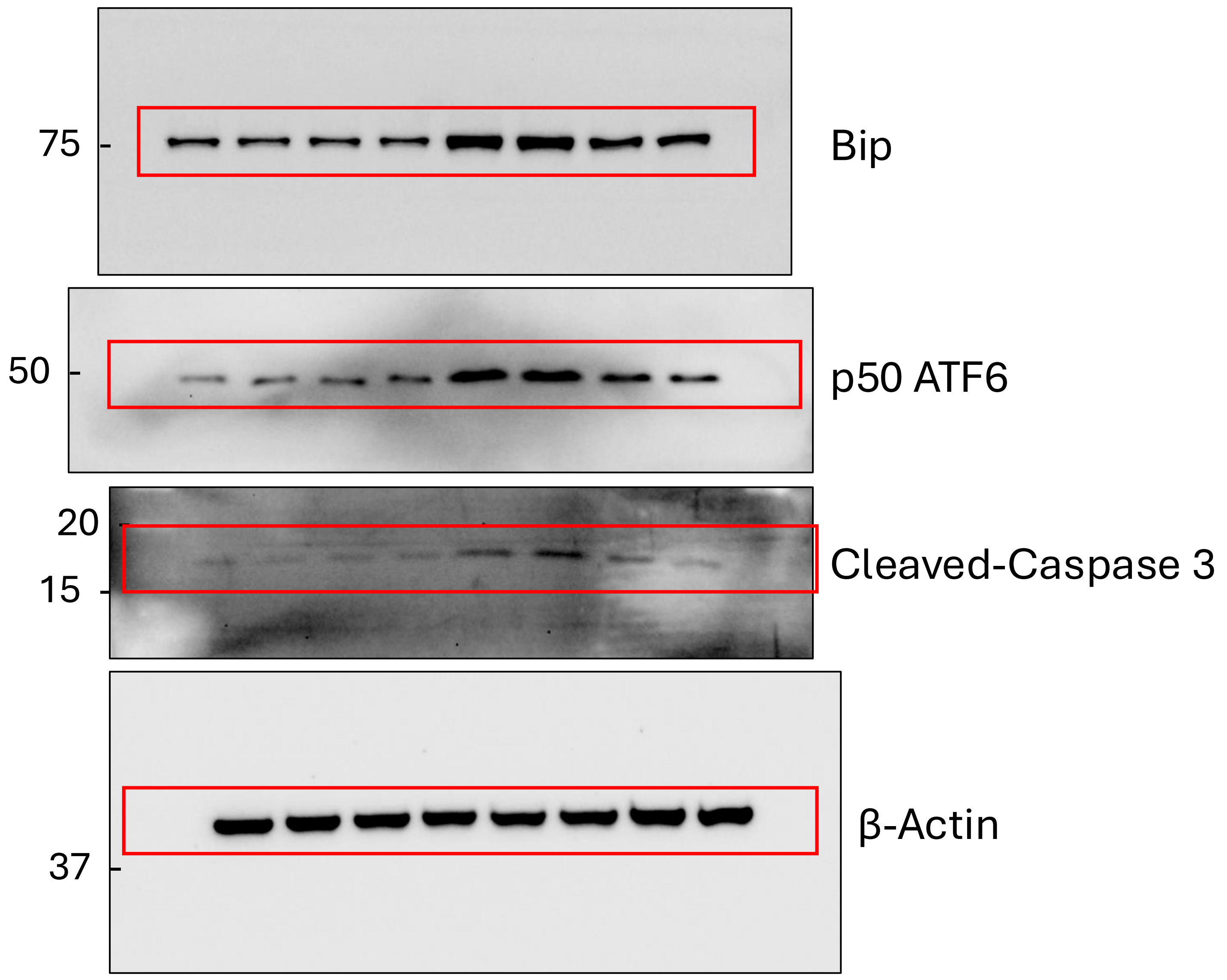

Supplement: Supplementary file 2 [file jci-136-205631-s294.pdf]
